# Associations between breastfeeding duration and overweight/obese among children aged 5–10: a focus on racial/ethnic disparities in California

**DOI:** 10.3934/publichealth.2019.4.355

**Published:** 2019-09-29

**Authors:** Christian E. Vazquez, Catherine Cubbin

**Affiliations:** Steve Hicks School of Social Work, The University of Texas at Austin, Austin, Texas, USA

**Keywords:** Hispanic, children, health disparities, breastfeeding, obesity

## Abstract

Research on the association between breastfeeding and childhood obesity and research on racial/ethnic differences in breastfeeding both show inconsistencies. The current study examines: 1) whether immigrant Hispanic women have higher rates of breastfeeding compared to non-Hispanic (three separate groups: African-American, Asian/Pacific Islander, and White) and U.S.-born Hispanic women; 2) whether children who were breastfed are less likely to be overweight/obese compared to children who were not breastfed; and 3) whether associations between breastfeeding and weight status vary by race/ethnicity/nativity. The study builds on prior literature using representative data from the Geographic Research On Wellbeing study (GROW, 2012–2013) and focusing on ages 5–10 years, an age group that has not been well studied (N = 2675 mother/child dyads). Logistic regression was used to investigate the odds of child obesity (≥95^th^%) and child overweight (≥85^th^%) in a series of models: unadjusted (each variable individually), demographic (child's sex, child's age, mother's age, mother's race/ethnicity, and mother's marital status), socioeconomic status (mother's education and family income), and full model (mother's BMI); with breastfeeding included in all models. Interactions between race/ethnicity and breastfeeding duration were also examined. African-American (9.54%) and white (32.8%) women had the lowest and highest rates of ever breastfeeding, respectively. White women breastfed the longest (M = 10.52 months, SE = 0.028) and U.S.-born Hispanic women breastfed the shortest (M = 7.05 months, SE = 0.41), on average. Children of African-American and U.S.-born Hispanic mothers had higher odds of being overweight/obese (74–75%) compared with children of white mothers. No associations were found between breastfeeding duration and child's weight status in adjusted models, nor was there a significant interaction between mother's race/ethnicity and breastfeeding duration on child's weight status; however, mother's own weight status was a significant driver of child's weight status and explained the racial/ethnic disparities. These results provide evidence in favor of there being no association between breastfeeding and childhood obesity.

## 1. Introduction

Recent studies reporting national childhood obesity rates in the United States suggest rates continue to be high with a steady increase in many populations [1]. One of the most prominent studies, using data from the National Health and Nutrition Examination Survey (NHANES), provided evidence that 18.5% of children were identified as obese in 2015–2016, the highest rates ever documented [1]. These rates are disproportionately higher for children of color, with recent reports showing rates as high as 25.8% and 22.0% for Hispanic and Black children, respectively, compared to White (14.0%) and Asian children (11.0%) [1],[2]. High rates of childhood obesity are a major concern considering the known health risks associated with being overweight/obese, such as developing type 2 diabetes, high blood pressure, heart disease, sleep apnea, liver disease, kidney disease, and certain types of cancer [1]. Children who are overweight/obese are also more likely to have negative non-health related outcomes, including poor educational outcomes and increased risk of being bullied [3]. The negative health risks can also be detrimental economically, with one study suggesting that being overweight/obese can lead to medical costs that are 42% higher than healthy-weight individuals [4].

Research on the association between breastfeeding and childhood obesity has resulted in inconclusive findings. Several recent studies and meta-analyses in the past decade have found evidence suggesting breastfeeding is a protective factor for childhood obesity [5]–[7]. Further, some studies suggest this is only true for certain groups, such as less acculturated Hispanic women or WIC participants [8]–[10]. There are also numerous studies that found no significant relationship between the two [11]–[13].

The lack of standardization of the way studies are being conducted may explain the variation of findings. The meta-analyses suffer from limitations due to small sample size and having to pool different types of breastfeeding measures (e.g., exclusive breastfeeding, mixed feeding, and exclusive formula feeding). Studies range from using ever breastfed to a specific number of weeks or months breastfed, which makes it difficult to compare studies. Studies are also difficult to compare because they use samples from prospective cohort, historical cohort, or cross sectional datasets. Another issue that affects all studies, including the meta-analyses, is the lack of standard covariates included across each study, with maternal BMI being a key covariate that is commonly missing. Further, only a few studies use representative samples. It is often the case that the study has no mention of race/ethnicity, or a small sample of racial/ethnic minorities. Alternatively, there are studies that focus on racial/ethnic minorities exclusively, primarily Hispanic populations, or a specific low-income population, which may bias results as these populations have overall higher rates of overweight/obesity. Additionally, the measure of height and/or weight varies across studies, from self-report to researcher measured. Lastly, a World Health Organization (WHO) report originally stated that breastfeeding was a protective factor for childhood obesity, but then rescinded that statement as further examination suggested there was evidence of publication bias [13],[14]. Yan et al. [6] also came to this conclusion, with their meta-analysis acknowledging that publication bias could not be ruled out, explaining that studies showing significant results are more likely to be published.

The American Academy of Pediatrics recommends breastfeeding as the optimal feeding practice during at least the first 6 months of the child's life based on the numerous advantages for both the mother and child, including developmental, economic, health, nutritional, and social benefits [15]–[17]. Despite these benefits, nearly 30% of infants in the U.S. are never breastfed [18],[19]. Based on the Healthy People 2020 objectives, Asian women are the only racial/ethnic group reaching the goal of breastfeeding initiation of 81.9%, although Hispanic women are close, with certain subgroups of Hispanic women (e.g. less acculturated, Mexican) meeting this goal [20]. Mothers with lower rates of breastfeeding tend to be young, with low incomes, unmarried, less educated, and overweight or obese before pregnancy [16],[20]. However, studies have found that immigrant Hispanic women with high breastfeeding rates tend to be anomalies with respect to some of the factors associated with not breastfeeding, as they tend to have lower incomes, be less educated, and have healthier pre-pregnancy weight, compared to all US-born mothers with similar breastfeeding rates [16],[21]. Alternatively, Wouk et al. [22] suggest Hispanic women tend to have low rates of breastfeeding, but did not examine differences among subgroups. Some studies have demonstrated that examining this relationship by subgroups of Hispanic populations is important because certain groups have different behaviors based on immigrant status or country of origin [16],[23].

Although various studies have examined the effects of breastfeeding as a protective factor for childhood obesity, few studies have investigated how this relationship may be different for racial/ethnic groups, especially minority families, using representative samples. Further, less attention has focused on children 5 to 10 years of age. The guiding question asks, if breastfeeding protects against childhood overweight/obesity, and immigrant Hispanic mothers have higher rates of breastfeeding (compared to non-Hispanics and US-born Hispanics), then are children born to immigrant Hispanic mothers less likely to be overweight/obese, compared to non-Hispanics and U.S.-born Hispanics? The purpose of the current study is to provide evidence on three fronts: 1) whether immigrant Hispanic women have higher rates of breastfeeding compared to non-Hispanic and U.S.-born Hispanic women; 2) whether children who were breastfed are less likely to be overweight/obese compared to children who were not breastfed; and 3) whether associations between breastfeeding and weight status varies by race/ethnicity/nativity.

## 2. Methods

### 2.1. Data source

Data for this study came from the Geographic Research On Wellbeing (GROW, 2012–2013) study. GROW is a population-based follow-up study of mothers interviewed at baseline for the California Maternal and Infant Health Assessment (MIHA), 2003–2007. MIHA, which is very similar to CDC's Pregnancy Risk Assessment Monitoring System, is an annual, statewide-representative mail/telephone survey of mothers delivering live infants in California, linked with birth certificate data (about 3500 respondents each year). Response rates for MIHA exceeded 70% each year from 2003 to 2007. Surveys were completed in English (71%) or Spanish (29%). Additional details about the MIHA survey are available [24]–[27].

Because of budget limitations, it was not feasible to follow-up all women for GROW who were interviewed at baseline. Therefore, a decision was made to follow-up MIHA respondents from six largely urbanized counties with the highest number of respondents. Respondents in these six counties represented 55% of all respondents in MIHA from 2003 to 2007. Women were eligible for GROW if they lived in one of these six counties and had agreed to be re-contacted for potential future studies (N = 9256). The GROW survey consisted of approximately 80 questions regarding demographic, socioeconomic, neighborhood, psychosocial, and health-related characteristics pertaining to herself and her index child aged 5 to 10 (her infant from the MIHA survey). The response rate was 75% among the sample of eligible women who were located (N = 3016 out of 4026 located). Over half completed the survey by phone, and nearly three-quarters completed it in English. Missing income values were imputed using hot-deck methodology and the following variables: age, race/ethnicity, education, employment status, marital status and neighborhood poverty. Weights were created to produce data that were representative of the birth file and original MIHA sample in the six GROW counties, and a sampling fraction file was created to make a minor finite population correction to the standard errors for analyses. Additional details about the GROW study are available [26].

The analytic dataset excluded women whose race/ethnicity was reported as American Indian/Alaska Native, missing, or “other” (N = 69). Other exclusions were children who did not live with the respondent at least half the time (N = 41) or whose current percentile of weight for their age was either missing (N = 204) or considered an outlier (N = 18) according to CDC standards. In addition, respondents were excluded if information was missing on breastfeeding (N = 59) resulting in 2675 records (89% of the GROW sample) for analysis.

The GROW study was approved by the Institutional Review Boards at the University of Texas at Austin, the University of California, San Francisco, and the California Department of Public Health; all participants gave informed consent.

### 2.2. Variables

The dependent variable was child's weight status. Mothers reported their child's weight in either pounds or kilos, which were converted to weight percentiles according to age, per CDC guidelines. Weight status was categorized as less than 85^th^ percent, 85^th^ to less than 95^th^ percent, and 95^th^ percent or higher. Above 85^th^ percent is considered overweight or obese, and above 95^th^ percent is considered obese. Due to height measurements being difficult for mothers to report, weight percentile rather than body mass index (BMI) was used. In a study evaluating the accuracy of parent reported height and weight, it was determined that parents more significantly underestimated height, particularly among younger children [28]. Additionally, the literature suggests that the use of weight percentiles as a proxy for BMI is acceptable if accurate height measurements are not available [29].

The primary independent variable was breastfeeding duration, measured in two ways. First, based on the literature indicating that greater than six months of breastfeeding is beneficial for healthy weight [5],[30] a categorical variable was created (none, less than seven months, or seven months or longer). Second, among women who ever breastfed (N = 2418), a continuous variable of the number of months of breastfeeding was created.

A number of sociodemographic factors were included as controls. These include child's sex, child's age, mother's age, mother's race/ethnicity (non-Hispanic African-American, non-Hispanic Asian/Pacific Islander, immigrant Hispanic, US-born Hispanic, and non-Hispanic White), mother's marital status, mother's education, family income, and mother's BMI (self-reported height and weight). Annual family income was measured as the total pretax income in 2011 from all sources. In combination with the number of people supported on that income, income was converted into increments of the federal poverty level.

### 2.3. Analysis

First, the distributions of covariates were examined. Next, bivariate relationships between breastfeeding duration or child weight status, and race/ethnicity were examined. Chi-square or t-tests were computed to compare proportions or means. Finally, logistic regression models were used to investigate the odds of child obesity (≥95^th^ percent) in a series of models: (a) unadjusted models (each variable individually); (b) a demographic model (child's sex, child's age, mother's age, mother's race/ethnicity, and mother's marital status); (c) a socioeconomic status model (demographic model plus mother's education and family income); (d) and a full model (socioeconomic status model plus Mother's BMI). All models were calculated with the categorical and continuous measure of breastfeeding, as well as with overweight or obese as the dependent variable (≥85^th^ percent), for a total of 4 iterations of the four models. Interactions between race/ethnicity and breastfeeding duration were also examined. All analyses were weighted, accounted for the complex sample design, and were conducted using SAS version 9.4.

## 3. Results

Table 1 presents descriptive characteristics of the sample. About half of the children were female and the average child's age was about seven years old. Mothers' average age was just under 29 years old, and 84% were married or living together. Over half of the mothers were Hispanic, followed by white, Asian/Pacific Islander, and African-American. About 60% of women had attended at least some college, and nearly half had family incomes that were at or below 200% of the federal poverty level. Twenty-three percent of mothers had BMIs that were in the obese range. While less than 10% of mothers did not breastfeed at all, nearly half breastfed for seven months or longer. Among women who ever breastfed, the average duration was nine months. Over three quarters of children had a normal weight status, compared with 13% who were overweight and nearly 11% who were obese.

Breastfeeding duration and child weight status varied significantly by race/ethnicity (Table 2). African-American women had the highest rate of no breastfeeding (21%), and white women had the lowest rate (6%). Among women who ever breastfed, U.S.-born Hispanic women breastfed the shortest amount of time (7 months) and white women breastfed the longest (10.5 months). Children of African-American or Hispanic mothers had the highest rates of being overweight/obese (26–27%), followed by children of white mothers (20%), and Asian/Pacific Islander mothers (17%).

Table 3 presents logistic regression models for obese children, using the categorical measure of breastfeeding duration. In unadjusted models, child's age, mother's race/ethnicity, education, income, mother's BMI, and breastfeeding duration were all significantly associated with child obesity. For example, children of mothers with less than high school education had 2.4 higher odds of reporting child obesity compared with children of mothers who were college graduates. After adjusting for child's sex, child's age, mother's age, mother's race/ethnicity, marital status and breastfeeding duration, only child's age and mother's race/ethnicity remained statistically significant. With additional adjustment for education and income, older children and children of African-American or U.S.-born Hispanic mothers had higher odds of reporting obesity compared with their reference groups. In the full model, with additional adjustment for mother's BMI, the only remaining significant effect was for children of obese mothers, whom had nearly 2.5 higher odds of obesity compared with children of normal weight mothers. No statistically significant interaction was found between mother's race/ethnicity and breastfeeding duration.

**Table 1.** Descriptive Characteristics, Geographic Research on Wellbeing Study (2012–2013), N= 2675 mother/child dyads.

	n (M)	% (SE)
Child's Sex		
Female	1292	49.1
Male	1383	50.9
Child's Age	(6.91)	(0.02)
Mother's Age	(28.78)	(0.13)
Mother's Race/ethnicity		
African-American	315	6.6
Asian/Pacific Islander	273	15.5
Hispanic – Immigrant	723	34.5
Hispanic – U.S. Born	425	16.5
White	939	26.9
Marital Status		
Married/living together	2228	83.9
Unmarried	435	16.1
Mother's Education		
< High School	402	18.4
High School Graduate/GED	477	22.3
Some College	654	23.6
College Graduate	1131	35.7
Family Income		
0–100 % FPL	581	28.5
101–200 % FPL	448	20.3
201–400 % FPL	509	20.5
>400 % FPL	898	30.7
Mother's BMI		
0–24.99	1213	45.7
25–29.99	774	31.0
30+	571	23.3
Breastfeeding Duration		
None	234	9.2
<7 months/none	1070	42.2
7 months +	1348	48.6
Breastfeeding Duration^a^ (months)	(9.33)	(0.18)
Child Weight Status		
<85^th^ %	2064	76.9
≥85^th^ – <95^th^ %	327	12.6
≥95^th^ %	284	10.5

Note: ^a^ Among ever breastfed (n = 2418). n(M) = sample size and mean. % (SE) = percent and standard error.

Nearly identical findings were observed using the continuous measure of breastfeeding duration among women who ever breastfed (Table 4). In models of child overweight/obese, similar results were also found (Appendix Tables 1–2). The main difference was that females had lower odds of being overweight/obese compared with male children and children of mothers who were either overweight or obese both had higher odds of being overweight/obese compared with children to mothers of normal weight.

**Table 2.** Breastfeeding Duration and Child Weight Status by Race/ethnicity, Geographic Research on Wellbeing Study (2012–2013), N = 2675 mother/child dyads.

	Race/ethnicity					
	African-American	Asian/Pacific Islander	Hispanic-Immigrant	Hispanic-U.S. Born	White	
	% or M (SE)	% or M (SE)	% or M (SE)	% or M (SE)	% or M (SE)	χ^2^ or t-test
Breastfeeding Duration						
None	21.4	6.4	7.0	14.8	5.9	
<7 months	42.6	43.6	43.0	53.4	35.5	67.5***
≥7 months	36.0	50.0	50.0	31.7	58.6	
						
Breastfeeding Duration^a^ (months)	8.46 (0.57)	9.96 (0.57)	9.19 (0.30)	7.05 (0.41)	10.51 (0.28)	301.8***
Child Weight Status						
<85^th^%	72.6	83.4	73.1	73.9	80.3	
≥85^th^ – <95^th^%	13.2	9.5	14.0	12.1	12.4	29.5***
≥95^th^%	14.2	7.1	12.9	14.0	7.3	

Note: *^a^* Among ever breastfed (n = 2418). % = percent. M = mean. SE = standard error. ***p < 0.001.

**Table 3.** Logistic Regression Models for ≥95^th^% by Categorical Breastfeeding, Geographic Research on Wellbeing Study (2012–2013), N = 2675 mother/child dyads.

	Unadjusted OR (95% CI)	Demographics OR (95% CI)	SES OR (95% CI)	Full OR (95% CI)
Child's Sex				
Female	0.79 (0.59–1.07)	0.77 (0.57–1.04)	0.76 (0.56–1.02)	0.76 (0.56–1.03)
Male	1.00	1.00	1.00	1.00
Child's Age	1.12 (1.02–1.23)*	1.11 (1.01–1.22)*	1.11 (1.01–1.23)*	1.10 (1.00–1.22)
Mother's Age	0.98 (0.96–1.01)	1.01 (0.98–1.03)	1.01 (0.99–1.04)	1.01 (0.99–1.04)
Mother's Race/ethnicity				
African-American	1.44 (0.96–2.16)	1.87 (1.14–3.08)*	1.75 (1.05–2.92)*	1.49 (0.89–2.51)
Asian/Pacific Islander	0.55 (0.32–0.93)*	0.87 (0.49–1.56)	0.88 (0.49–1.59)	0.92 (0.51–1.67)
Hispanic-Immigrant	1.43 (1.05–1.95)*	1.88 (1.29–2.73)**	1.40 (0.86–2.27)	1.34 (0.82–2.20)
Hispanic-U.S. Born	1.45 (1.02–2.05)*	1.97 (1.27–3.06)**	1.74 (1.11–2.72)*	1.58 (1.00–2.50)
White	1.00	1.00	1.00	1.00
Marital Status				
Married/living together	1.00	1.00	1.00	1.00
Unmarried	1.39 (0.99–1.97)	1.18 (0.81–1.71)	1.14 (0.77–1.68)	1.15 (0.78–1.70)
Mother's Education				
<High School	2.37 (1.57–3.58)***		1.77 (0.99–3.17)	1.54 (0.85–2.79)
High School Graduate/GED	1.75 (1.17–2.62)**		1.28 (0.74–2.24)	1.15 (0.67–2.00)
Some College	1.49 (1.02–2.17)*		1.12 (0.72–1.73)	1.03 (0.66–1.61)
College Graduate	1.00		1.00	1.00
Family Income				
0–100 % FPL	2.06 (1.40–3.05)***		1.18 (0.67–2.09)	1.03 (0.58–1.82)
101–200 % FPL	2.00 (1.32–3.01)**		1.30 (0.77–2.20)	1.16 (0.68–1.98)
201–400 % FPL	1.94 (1.29–2.92)**		1.55 (0.99–2.43)	1.45 (0.92–2.28)
> 400 % FPL	1.00		1.00	1.00
Mother's BMI				
0–24.99	1.00			1.00
25–29.99	1.78 (1.24–2.57)**			1.47 (1.00–2.17)
30+	3.12 (2.17–4.47)***			2.45 (1.66–3.63)*
Breastfeeding Duration				
None	1.75 (1.09–2.78)*	1.44 (0.89–2.35)	1.31 (0.79–2.17)	1.28 (0.77–2.13)
<7 months	1.37 (1.00–1.87)	1.26 (0.90–1.76)	1.21 (0.86–1.70)	1.15 (0.82–1.62)
7 months +	1.00	1.00	1.00	1.00

Note: Models: (a) unadjusted models (each variable individually); (b) demographic model (child's sex, child's age, mother's age, mother's race/ethnicity, and mother's marital status); (c) socioeconomic status model (demographic model plus mother's education and family income); (d) and full model (socioeconomic status model plus Mother's BMI); all models include breastfeeding duration (categorical variable). * p < 0.05. ** p < 0.01. *** p < 0.001.

**Table 4.** Logistic Regression Models for ≥95^th^% by Continuous Breastfeeding, Geographic Research on Wellbeing Study (2012–2013), N = 2418 mother/child dyads.

	Unadjusted OR (95% CI)	Demographics OR (95% CI)	SES OR (95% CI)	Full OR (95% CI)
Child's Sex				
Female	0.80 (0.60–1.08)	0.73 (0.53–1.01)	0.72 (0.52–1.00)*	0.73 (0.52–1.01)
Male	1.00	1.00	1.00	1.00
Child's Age	1.12 (1.02–1.23)*	1.13 (1.02–1.25)*	1.14 (1.02–1.26)*	1.12 (1.01–1.24)*
Mother's Age	0.98 (0.96–1.01)	1.00 (0.98–1.03)	1.01 (0.98–1.04)	1.01 (0.98–1.04)
Mother's Race/ethnicity				
African-American	1.48 (0.99–2.22)	2.00 (1.15–3.48)*	1.83 (1.04–3.21)*	1.57 (0.88–2.80)
Asian/Pacific Islander	0.55 (0.32–0.93)*	1.02 (0.56–1.85)	1.05 (0.58–1.91)	1.09 (0.59–2.01)
Hispanic-Immigrant	1.41 (1.04–1.92)*	2.04 (1.37–3.02)***	1.51 (0.90–2.54)	1.48 (0.87–2.51)
Hispanic-U.S. Born	1.45 (1.03–2.05)*	2.03 (1.26–3.26)**	1.78 (1.09–2.91)*	1.58 (0.94–2.66)
White	1.00	1.00	1.00	1.00
Marital Status				
Married/living together	1.00	1.00	1.00	1.00
Unmarried	1.40 (0.99–1.97)	1.31 (0.89–1.95)	1.27 (0.85–1.91)	1.28 (0.85–1.94)
Mother's Education				
<High School	2.38 (1.58–3.58)***		1.83 (0.98–3.39)	1.56 (0.82–2.95)
High School Graduate/GED	1.75 (1.17–2.61)**		1.17 (0.63–2.18)	1.06 (0.57–1.96)
Some College	1.51 (1.04–2.19)*		1.14 (0.73–1.80)	1.08 (0.68–1.71)
College Graduate	1.00		1.00	1.00
Family Income				
0–100% FPL	2.05 (1.40–3.03)***		1.23 (0.66–2.27)	1.03 (0.55–1.91)
101–200% FPL	1.97 (1.31–2.97)**		1.42 (0.81–2.49)	1.23 (0.70–2.17)
201–400% FPL	1.89 (1.26–2.84)**		1.66 (1.04–2.64)*	1.52 (0.95–2.45)
>400% FPL	1.00		1.00	1.00
Mother's BMI				
0–24.99	1.00			1.00
25–29.99	1.77 (1.23–2.55)**			1.42 (0.94–2.15)
30+	3.08 (2.15–4.41)***			2.67 (1.77–4.02)***
Breastfeeding duration^a^	0.99 (0.97–1.01)	1.00 (0.98–1.02)	1.00 (0.98–1.02)	0.99 (0.92–1.06)

Note: ^a^ Among ever breastfed. Models: (a) unadjusted models (each variable individually); (b) demographic model (child's sex, child's age, mother's age, mother's race/ethnicity, and mother's marital status); (c) socioeconomic status model (demographic model plus mother's education and family income); (d) and full model (socioeconomic status model plus Mother's BMI); all models include breastfeeding duration (continuous variable). * p < 0.05. ** p < 0.01. *** p < 0.001.

## 4. Discussion

The purpose of the current study is to provide evidence on three fronts: 1) whether immigrant Hispanic women have higher rates of breastfeeding compared to non-Hispanic and U.S.-born Hispanic women; 2) whether children who were breastfed are less likely to be overweight/obese compared to children who were not breastfed; and 3) whether associations between breastfeeding and weight status varies by race/ethnicity/nativity. As discussed above, the literature on the topics of breastfeeding rates and breastfeeding as a protective factor against obesity has rendered mixed results. The results from this study provide evidence in favor of one side of the debate related to each topic.

In this sample, white women had the lowest rates of having never breastfed, and breastfed the longest, with immigrant Hispanic women and Asian/Pacific Islander women close behind. In contrast, African-American women and U.S.-born Hispanic women had the highest rates of never breastfeeding and breastfed the shortest amount of time. The finding that African-American women and U.S.-born Hispanic women tend to have the worst outcomes is consistent throughout the literature [18],[19]. Research has document that high-acculturated Hispanic women are less likely to intend to or breastfeed their newborn, compared with low-acculturated Hispanic women [23]. One possible explanation for this is that African-American, U.S.-born Hispanic, and immigrant Hispanic women tend to share low-income status; however, immigrant Hispanic women also tend to stay at home instead of joining the labor force, which makes it easier to have time to breastfeed. Another explanation may be the normality of using formula in the United States. Through programs, such as Women, Infants, and Children (WIC), formula is readily available and is commonly used, whereas postnatal resources in South and Central America are not as available and use of formula is not common practice [16]. Additionally, the literature provides evidence that the low prevalence of exclusive breastfeeding in low-resourced countries may be due to caregivers introducing solid food at earlier stages of infancy [31]. Further, in a review examining the relationship between childhood obesity and breastfeeding among African-Americans, barriers to breastfeeding included impact on personal and family life, including going back to work, a lack of social support, fear of impact on sexuality, fear of pain, and lack of education [32]. One of the biggest barriers to increasing breastfeeding among African-American and U.S.-born Hispanic women continues to be education opportunities, attributed to both cultural relevance of information and convenience of location to receive the information [20],[33]. Concerted efforts have been made to address these issues; yet the barriers persist. This leaves a gap for innovative approaches. One such policy that is attempting to fill this gap is the increase in Baby-Friendly hospitals [34]. Baby-Friendly hospitals are designed to optimize mother-baby bonding and to protect, promote and support breastfeeding in the first few days of a new baby's life [34]. There are currently around 500 hospitals in the United States with this designation [34]. However, the societal impact of this policy has yet to be evaluated.

This study's results also indicated that children of immigrant Hispanic women had higher odds of being overweight/obese compared to children of non-Hispanic white women; however, that difference was no longer statistically significant after controlling for education and income. Moreover, breastfeeding was not found to be a protective factor against overweight/obesity for any race/ethnicity (i.e., no significant interaction effect between race/ethnicity and breastfeeding). After adjustment for demographic and socioeconomic factors, African-American children and those of U.S.-born Hispanic women have about a 75% higher odds of obesity. The mechanism explaining the remaining racial/ethnic disparity is through mothers' own BMI. Literature spanning over two decades provides evidence that mother's BMI continues to be a strong predictor of childhood overweight/obesity status [10],[35]. Not surprisingly, research in the area of childhood obesity interventions has moved toward studying the engagement of parents as part of the weight reduction program, as this has demonstrated successful outcomes for both the parent and child [36]. As the debate on whether or not breastfeeding is a protective factor against childhood overweight/obesity continues, the current study provides evidence in favor of there being no association between the two. This also provides evidence for possible publication bias, discussed in previous studies [13],[14].

The Hispanic Paradox is the phenomenon that immigrant Hispanics are healthier than U.S.-born Hispanics and non-Hispanics, despite very low socioeconomic status [37]. Several studies, including Baker et al. [21] set out to understand if the Hispanic Paradox extends from caregivers to Hispanic children [38]. They found that after a certain amount of years any health benefits children were receiving from having a healthy immigrant parent had disappeared [38]. Previous studies suggest obesity, in particular, is a health issue that appears quicker than other health issues because of the decline in healthy eating at a young age [39],[40]. The results of the current study are supportive of the Paradox not protecting children of immigrants, given that children of immigrant Hispanic women have higher odds of obesity, compared to children of non-Hispanic white women.

Children born to all Hispanic or African-American caregivers are disproportionately at-risk when it comes to obesogenic behaviors, compared to non-Hispanic whites [41],[42]. For example, healthier dietary habits are more common in families that are better educated, have a higher household income, and have a lower parental BMI, which is more common for white and Asian caregivers [40],[43]. Studies examining dietary habits among Hispanics and groups of African origin at various ages found greater levels of acculturation to be associated with more unhealthy dietary habits, such as greater intakes of salty snacks and energy-dense foods and lower intake of fruit and vegetables [40],[44]. Similarly, physical inactivity levels, an important behavioral determinant of childhood obesity risk [45], are highest among Hispanic and African-American groups, and increase with longer duration in the U.S. [46]. Physical activity disparities place African-American and Hispanic youth at higher risk for diabetes, obesity, and cardiovascular disease [47]–[49].

### 4.1. Strengths and limitations

The inability to examine differences among Hispanic subgroups is a limitation of the current study but the finding that immigrant Hispanic women had breastfeeding rates similar to white women provides some evidence that immigrant subgroup differences may be driving these higher rates. It is important for future studies to examine Hispanic subgroup differences to the extent possible because the information will help public health practitioners and policy-makers identify which groups to target and what interventions might be culturally relevant to each group. Examining subgroups is a challenge with many datasets due to the low sample size across groups, but should be attempted whenever possible. Given that 81.5% of Hispanics in California are Mexican origin, the current study's results mostly reflect that group [50]. Duration in the U.S. and language, other important indicators for understanding immigrant health behaviors, was also not included. Another limitation is that this study used weight for age percentile based on mother's report of weight, which some argue gives inaccurate estimates [51]; however, another study suggests this practice is acceptable if accurate height measurements are not available [29]. Lastly, the current study does not account for possible neighborhood effects, such as safety concerns (e.g., reported crime), the built environment (e.g., lack of parks and playgrounds), and presence or lack of neighborhood resources (e.g., grocery stores), which may be a factor in mother's and child's weight status.

Despite the limitations, the current study has several strengths. The GROW study is a representative sample from a large and diverse state. This study helps to fill a gap in the literature by examining the relationship between breastfeeding and obesity outcomes for children between 5 to 10 years of age. Sub-groups of Hispanic women according to immigrant status were also examined.

### 4.2. Implications and future research

Based on the findings of the present study, it is suggested that interventions to increase breastfeeding provide tailored supports to U.S.-born Hispanic and African-American mothers, as they tend to experience disproportionately lower levels of breastfeeding. Given high levels of overweight and obesity overall, interventions promoting healthier weight for young children of all race/ethnicities in general may benefit from including components to enhance family involvement to simultaneously reduce parental BMI. Future studies should examine cultural, occupational, and social support factors that may be barriers to breastfeeding. Future research should also expand to explore effects of neighborhood level factors. The answers to these questions have implications for all persons attempting to increase breastfeeding rates and reduce racial/ethnic disparities in weight status among mothers and children by aiding the development of interventions that target familial or neighborhood level factors. Additionally, it is important to investigate the effect of changes in laws and policies, health promotion, the Special Supplemental Program for Women, Infants, and Children, and employer support [52].

## 5. Conclusion

The present study sought out to provide evidence to clarify previous mixed findings on the relationship between breastfeeding and children's weight status. No associations were found between breastfeeding duration and child's weight status in adjusted models, nor was there a significant interaction between mother's race/ethnicity and breastfeeding duration on child's weight status; however, mother's own weight status was a significant driver of child's weight status and explained the racial/ethnic disparities. These results provide evidence in favor of there being no association between breastfeeding and childhood weight status.
