# Supplementary material for: Associations between breastfeeding duration and overweight/obese among children aged 5–10: a focus on racial/ethnic disparities in California
Source: AIMS Public Health. 2019 Sep 29;6(4):355–69. doi: 10.3934/publichealth.2019.4.355 (PMC6940568; doi:10.3934/publichealth.2019.4.355)
Supplement: Supplementary file 1 [file publichealth-06-04-355-s001.pdf]

AIMS Public Health

AIMS Public Health, 6(4): 355–369.
DOI: 10.3934/publichealth.2019.4.355
Received: 09 May 2019
Accepted: 23 September 2019
Published: 29 September 2019

http://www.aimspress.com/journal/aimsph

Research article

Associations between breastfeeding duration and overweight/obese among children aged 5–10: a focus on racial/ethnic disparities in California

Christian E. Vazquez* **and Catherine Cubbin**

Steve Hicks School of Social Work, The University of Texas at Austin, Austin, Texas, USA

* **Correspondence:** Email: christian.vazquez@utexas.edu; Tel: +8052596963.

Supplementary

Appendix 1. Logistic Regression Models for ≥85th% by Categorical Breastfeeding, Geographic Research on Wellbeing Study (2012–2013), N = 2675 mother/child dyads.

	Unadjusted OR (95% CI)	Demographics OR (95% CI)	SES OR (95% CI)	Full OR (95% CI)
Child's Sex				
Female	0.74 (0.59–0.91)**	0.72 (0.58–0.90)**	0.71 (0.57–0.89)**	0.71 (0.57–0.89)**
Male	1.00	1.00	1.00	1.00
Child's Age	1.13 (1.05–1.21)***	1.13 (1.05–1.21)***	1.13 (1.05–1.22)***	1.12 (1.04–1.21)**
Mother's Age	0.99 (0.97–1.00)	1.00 (0.98–1.02)	1.01 (0.99–1.03)	1.01 (0.99–1.03)
Mother's Race/ethnicity				
African-American	1.41 (1.03–1.93)*	1.62 (1.11–2.37)*	1.54 (1.05–2.28)*	1.35 (0.91–2.01)
Asian/Pacific Islander	0.58 (0.40–0.85)**	0.80 (0.53–1.21)	0.81 (0.53–1.22)	0.84 (0.56–1.28)
Hispanic-Immigrant	1.40 (1.11–1.77)**	1.58 (1.20–2.09)**	1.22 (0.84–1.78)	1.17 (0.80–1.70)
Hispanic-U.S. Born	1.21 (0.92–1.59)	1.50 (1.08–2.09)*	1.36 (0.97–1.92)	1.25 (0.88–1.76)
White	1.00	1.00	1.00	1.00
Marital Status				
Married/living together	1.00	1.00	1.00	1.00
Unmarried	1.34 (1.02–1.76)*	1.17 (0.87–1.56)	1.12 (0.83–1.52)	1.14 (0.84–1.54)
Mother's Education				
<High School	1.88 (1.38–2.57)***		1.54 (0.99–2.40)	1.36 (0.86–2.14)
High School Graduate/GED	1.65 (1.23–2.21)***		1.35 (0.90–2.02)	1.24 (0.82–1.86)
Some College	1.26 (0.96–1.66)		1.06 (0.78–1.44)	0.99 (0.72–1.36)
College Graduate	1.00		1.00	1.00
Family Income				
0–100 % FPL	1.75 (1.32–2.31)***		1.12 (0.73–1.73)	0.99 (0.64–1.53)
101–200 % FPL	1.60 (1.18–2.18)**		1.15 (0.78–1.69)	1.04 (0.70–1.53)
201–400 % FPL	1.46 (1.09–1.98)*		1.25 (0.91–1.74)	1.18 (0.85–1.64)
>400 % FPL	1.00		1.00	1.00
Mother's BMI				
0–24.99	1.00			1.00
25–29.99	1.81 (1.40–2.34)***			1.59 (1.21–2.09)***
30+	2.64 (2.02–3.44)***			2.25 (1.68–3.01)***
Breastfeeding Duration				
None	1.34 (0.92–1.94)	1.14 (0.77–1.69)	1.05 (0.71–1.57)	1.03 (0.69–1.53)
<7 months	1.14 (0.91–1.43)	1.06 (0.84–1.35)	1.04 (0.81–1.32)	0.99 (0.78–1.26)
7 months +	1.00	1.00	1.00	1.00

Note: Models: (a) unadjusted models (each variable individually); (b) demographic model (child's sex, child's age, mother's age, mother's race/ethnicity, and mother's marital status); (c) socioeconomic status model (demographic model plus mother's education and family income); (d) and full model (socioeconomic status model plus Mother's BMI); all models include breastfeeding duration (categorical variable). * $p < 0.05$. ** $p < 0.01$. *** $p < 0.001$.

Appendix 2. Logistic Regression Models for ≥85th% by Continuous Breastfeeding, Geographic Research on Wellbeing Study (2012–2013), N = 2418 mother/child dyads.

	Unadjusted OR (95% CI)	Demographics OR (95% CI)	SES OR (95% CI)	Full OR (95% CI)
Child's Sex				
Female	0.75 (0.60–0.93)*	0.71 (0.57–0.90)**	0.71 (0.56–0.89)**	0.72 (0.57–0.90)**
Male	1.00	1.00	1.00	1.00
Child's Age	1.14 (1.06–1.22)***	1.15 (1.07–1.24)***	1.16 (1.07–1.25)***	1.14 (1.06–1.24)***
Mother's Age	0.99 (0.97–1.00)	1.00 (0.98–1.02)	1.00 (0.98–1.03)	1.01 (0.98–1.03)
Mother's Race/ethnicity				
African-American	1.41 (1.03–1.93)*	1.77 (1.17–2.67)**	1.67 (1.10–2.55)*	1.47 (0.98–2.26)
Asian/Pacific Islander	0.58 (0.40–0.85)**	0.91 (0.60–1.38)	0.91 (0.60–1.39)	0.94 (0.62–1.44)
Hispanic-Immigrant	1.41 (1.12–1.79)**	1.68 (1.25–2.24)***	1.30 (0.87–1.94)	1.26 (0.84–1.89)
Hispanic-U.S. Born	1.20 (0.92–1.58)	1.52 (1.06–2.16)*	1.39 (0.96–2.00)	1.27 (0.85–1.61)
White	1.00	1.00	1.00	1.00
Marital Status				
Married/living together	1.00	1.00	1.00	1.00
Unmarried	1.32 (1.01–1.73)*	1.21 (0.89–1.64)	1.16 (0.85–1.59)	1.17 (0.85–1.61)
Mother's Education				
<High School	1.95 (1.43–2.66)***		1.52 (0.94–2.43)	1.30 (0.80–2.13)
High School Graduate/GED	1.65 (1.23–2.21)***		1.25 (0.81–1.94)	1.14 (0.73–1.79)
Some College	1.27 (0.97–1.67)		1.02 (0.74–1.41)	0.96 (0.69–1.34)
College Graduate	1.00		1.00	1.00
Family Income				
0–100 % FPL	1.74 (1.32–2.30)***		1.18 (0.75–1.88)	1.03 (0.64–1.65)
101–200 % FPL	1.61 (1.18–2.18)**		1.16 (0.77–1.74)	1.03 (0.68–1.56)
201–400 % FPL	1.47 (1.09–1.98)*		1.34 (0.96–1.87)	1.26 (0.90–1.77)
>400 % FPL	1.00		1.00	1.00
Mother's BMI				
0–24.99	1.00			1.00
25–29.99	1.78 (1.38–2.31)***			1.53 (1.15–2.03)**
30+	2.58 (1.98–3.37)***			2.30 (1.69–3.13)***
Breastfeeding duration[a]	1.00 (0.98–1.01)	1.00 (0.99–1.01)	1.00 (0.99–1.02)	1.00 (0.99–1.02)

Note. [a] Among ever breastfed. Models: (a) unadjusted models (each variable individually); (b) demographic model (child's sex, child's age, mother's age, mother's race/ethnicity, and mother's marital status); (c) socioeconomic status model (demographic model plus mother's education and family income); (d) and full model (socioeconomic status model plus Mother's BMI); all models include breastfeeding duration (continuous variable). * $p < 0.05$. ** $p < 0.01$. *** $p < 0.001$.

AIMS Press

©2019 the Author(s), licensee AIMS Press. This is an open access article distributed under the terms of the Creative Commons Attribution License (http://creativecommons.org/licenses/by/4.0)
